# Exploring representations of human grasping in neural, muscle and kinematic signals

**DOI:** 10.1038/s41598-018-35018-x

**Published:** 2018-11-12

**Authors:** Andreea I. Sburlea, Gernot R. Müller-Putz

**Affiliations:** Institute of Neural Engineering, Graz University of Technology, Graz, Austria

## Abstract

Movement covariates, such as electromyographic or kinematic activity, have been proposed as candidates for the neural representation of hand control. However, it remains unclear how these movement covariates are reflected in electroencephalographic (EEG) activity during different stages of grasping movements. In this exploratory study, we simultaneously acquired EEG, kinematic and electromyographic recordings of human subjects performing 33 types of grasps, yielding the largest such dataset to date. We observed that EEG activity reflected different movement covariates in different stages of grasping. During the pre-shaping stage, centro-parietal EEG in the lower beta frequency band reflected the object’s shape and size, whereas during the finalization and holding stages, contralateral parietal EEG in the mu frequency band reflected muscle activity. These findings contribute to the understanding of the temporal organization of neural grasping patterns, and could inform the design of noninvasive neuroprosthetics and brain-computer interfaces with more natural control.

## Introduction

The pathway between the brain and the hand, which ultimately enables the motor control of reaching and grasping movements, has been of interest to scientists for many decades^1–7^. So far, most neural decoders of grasping movements have been developed to control neuroprosthetic devices or robotic limbs. These decoders have been evaluated invasively in non-human primates^1,8,9^ and in humans with sensorimotor disabilities^5,10–14^, but also noninvasively in humans^15–24^. Typically, such neural decoders rely on optimizing the level of discriminability between the (grasping) movements. However, knowledge is still lacking about how the neural mechanisms behind the neural decoders of distinct grasping movements are represented in electroencephalography (EEG), and how these EEG representations are related to the behavioral response described by covariates of movements such as those that result from muscle or kinematic activity.

To gain a better understanding of the mechanisms that the brain uses to conduct goal-directed movements, researchers seek and evaluate representational models^25^ that can capture differences between movements by comparing behavioral movement features with neural patterns^26–29^. Such models can be used to address not only the relation between the movement patterns encoded in specific brain regions, but also the extent to which the neural representation of the movement is reflected in the patterns of different movement covariates.

We believe that gaining an understanding of the underlying neural functions used by the brain to perform grasping movements can augment the knowledge revealed by neural decoding performance. Some studies have addressed the difference between the brain patterns that led to high decoding performance and the underlying brain processes that are necessary to accomplish a task^30,31^. For example, the findings of several studies have shown that the role of primary motor cortex is to generate movement but not represent it^32,33^. It is well established in movement neuroscience that the primary motor cortex is one of the main sources of cortical output to the spinal cord during reach-to-grasp movements^34–36^. However, other regions like dorsal and ventral premotor cortices, as well as the posterior parietal cortex have also been associated with reach-to-grasp movements^37–40^. Furthermore, a recent study that attempted to characterize the representation of sequences of finger movements using functional magnetic resonance imaging (fMRI) recordings^29^ showed that premotor and parietal areas encode sequences of finger movements. Such behavior can be observed in dexterous movements such as grasping movements. Moreover, the findings of these studies have supported the existence of a temporal relation between different stages of grasping movements that involve similar brain areas but at different time points.

Grasping is a complex, skilled behavior that consists of several temporal movement stages, beginning with the planning of the movement and ending with the finalization of the grasping execution. The hand pre-shaping stage depends on the object’s intrinsic properties, such as its size and shape. The hand pre-shapes during its journey to the final destination (i.e., the target object)^41^. The generation and representation of reach-to-grasp movements involve distinct brain regions. Acquiring neural data using EEG benefits from a high temporal resolution and allows us to record from broad brain areas, capturing the interactions between the different regions involved in the prehension network^42–47^. The findings of previous studies have revealed differences in the EEG neural correlates of different movements of the same arm/hand (e.g., supination/pronation, opening/closing)^48^, as well as among three grasp types: power, pincer and intermediate grasps^21,49^. However, it is still unclear whether different brain areas and frequency bands are involved in different stages of the grasping movement, and which covariates of the grasping movement are encoded in these brain patterns.

We believe that exploratory approaches in the case of broad research questions are as relevant as the confirmatory approaches for specific, narrowed down questions^50,51^. Hence, in this exploratory study, we chose to investigate the similarity in human grasping movements between EEG representations and their associated movement covariates, in three stages of the grasping movement: hand pre-shaping, reach of the final grasping position and holding. Having in mind this exploratory goal for our investigation, we recorded a rich dataset containing simultaneous EEG, electromyographic (EMG) and kinematic activity, while 31 human subjects performed 33 different types of grasping movements, yielding the largest such dataset to date. We categorized the grasps based on the shape and size of the object, position of the thumb relative to the palm, in addition to the standard grasp type categorization in power, pincer and intermediate. We conducted this study at a group level, and applied representational similarity analysis (RSA)^52–54^ to investigate the similarities among the effects of the neural, behavioral (muscle and kinematic) representations and categorical models at different stages of grasping movements.

## Materials and Methods

### Participants

We recruited 31 healthy human participants (15 females and 16 males, mean age 25.2 ± 3.4 years old). All participants had normal or corrected-to-normal vision and had no reported neurological or musculoskeletal disorders. All subjects were right-handed. In addition, we also investigated their handedness through an adapted version of the Edinburgh Handedness Inventory^55^, which is used to evaluate the hand preference for several grasping types. We have included the exact items of the questionnaire in the Supplementary Materials (Appendix A). This experiment conformed with the statements of the Declaration of Helsinki (DoH) regarding ethical principles for medical research involving human participants. In addition, the protocol of this study was approved by the ethical committee of Medical University of Graz (approval number: 29–352 ex16/17). After being briefed regarding the protocol of the study, participants signed the informed consent. They were paid 7.5 euros per hour for their participation.

### Experimental task

The experimental task required the execution of 33 different grasp types, which are described in more detail in the Supplementary Materials (Appendix B). We chose this set of grasps because they are part of a taxonomy of grasps^56^ that contains the most thoroughly investigated collection of static and stable grasps performed with one hand. We categorized the grasps based on three criteria: type of grasp, position of the thumb relative to the palm and object’s shape and size.

**Figure 1:**
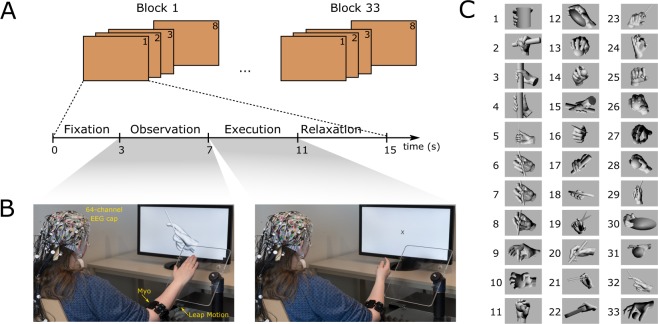
(**A**) Experimental protocol. Each of the 33 blocks contained eight consecutive repetitions (trials) of the same grasp. Each trial had four phases: fixation (three seconds long), observation (four seconds long), execution (four seconds long) and relaxation (four seconds long). (**B**) Experimental setup. Photos of one participant during the observation and execution phases, and the materials used during recording. (**C**) Pictograms of the grasping movements.

First, participants attended a practice session at which they became familiar with each of the 33 grasps that they would have to perform later in the study. To this purpose, we presented one example of each of the 33 pictures of the grasps and instructed the participants to explore the position of the fingers, as well as the shape and size of the object, shown in an image on the computer screen located in front of them. Next, the participants were asked to replicate as closely as possible the observed grasp using their right hand, but without using any object. After attending the practice session, subjects took a short break and then took part in the actual experimental task. Each of the 33 grasps was presented in blocks of eight sequential repetitions. Each repetition (trial) was fifteen seconds long. The structure of a trial as a part of a block is depicted in Fig. 1A.

First, as part of a trial, participants were instructed to focus their gaze on a cross located in the middle of the screen and avoid eye movements for three seconds. Next, during the observation phase which lasted four seconds, participants were presented with a static image showing a hand in a final grasping position together with the grasped object. During this observation phase, participants could move their eyes and observe the shape and size of the object, as well as the position of the fingers and the overall shape of the hand. During the fixation and observation phases, as well as during the break, participants rested their hand on a transparent, custom-made plexiglass hand support, shown on the right side in Fig. 1B. We built this hand support to reduce hand fatigue and record the resting position using the Leap Motion optical tracking device. During the execution phase which was also four seconds long, participants were instructed to focus their gaze on the “x” symbol located in the middle of the screen and to lift their hand from the resting support, move it slightly to the left and perform the grasping movement that they had observed during the previous phase. The acquisition of the blocks was divided in 4 parts. Each of the first three parts contained 8 blocks and lasted 16 minutes, while the last part contained 9 blocks. After each part, we offered longer breaks to adjust the needs of the participants. The total duration of one recording was on average an hour and half. Fig. 1C shows the pictograms of the 33 grasping conditions together with their ordinal numbers. The order of the grasping conditions (blocks) was randomized among subjects. The participant shown in Fig. 1C gave her informed consent for the photo to be made available in an open-access publication.

### Data acquisition

For the simultaneous acquisition of the data, we used the Lab Streaming Layer (LSL) framework (https://github.com/sccn/labstreaminglayer). For the recording of the EEG and EOG activity, we used a 64-channel ActiCap system connected to two 32-channel BrainAmp amplifiers (BrainProducts, Germany). The data was sampled at 1 kHz. The amplifiers have a hardware first-order, high-pass filter operating at 0.01 Hz and a low-pass filter operating at 250 Hz. EEG activity was recorded through 61 channels covering all the brain regions according to the 10-10 international system. The remaining three channels were used to record EOG activity from the outer canthi of the left and right eyes and from the above of the nasion. The ground sensor was placed on AFz and the reference sensor on the right mastoid. To record the muscle activity, we used a Myo armband (Thalmic Labs Inc., Ontario, Canada). This device has eight equidistant EMG sensors and a nine-axis inertial measurement unit (IMU) which provides accelerometer, gyroscope and magnetometer data. The data was acquired via the Bluetooth Low Energy protocol. The Myo armband requires calibration every time it is put on the arm. We only calibrated the armband at the beginning of the experiment, since none of the participants removed the armband during the study. The armband was located on the right arm close to the elbow, above the extrinsic hand muscles. It has been found that the reconstruction of the grasps of different-sized objects can be obtained using only extrinsic muscles^57,58^. Hence, we targeted the extrinsic muscles of the hand such as the palmaris longus, flexor carpi radialis, brachioradialis, and flexor carpi ulnaris muscles on the anterior forearm, and the extensor carpi ulnaris, extensor digitorum, extensor carpi radialis brevis, extensor carpi radialis longus muscles on the posterior forearm. EMG activity was recorded at a sampling rate of 200 Hz. A hardware filter was applied to remove powerline interference and then, the EMG activity was streamed through 8 channels of 8-bit data. The other signals from the Myo armband were recorded at 50 Hz.

To record the kinematics related to the grasping movements, we used the Leap Motion controller (Leap Motion Inc., San Francisco, CA, USA). Using a customized application for the LSL, we recorded kinematic data using the Leap Motion controller on the finger segments length, position and joint angles, as well as the palm position and velocity. These were recorded at a variable sampling rate between 80 and 120 Hz. Later in the analysis, we interpolated the data using a cubic spline interpolation, to achieve a fixed sampling rate of 100 Hz. The timestamps of the grasp’s presentation were synchronized offline with the other recorded signals by means of a photodiode, which captured an impulse on the screen at the start of each observation period.

### Data preprocessing

For all data preprocessing and analyses, we used Matlab R2016b (Mathworks, Inc. USA). EEG data was first filtered using a Butterworth fourth-order, zero-phase, band-pass filter between 0.1–40 Hz and then downsampled to 100 Hz. EEG, EMG and joint angles data were epoched in fifteen-second-long segments relative to the beginning of the trial. All the trials were reordered according to a common order of grasping conditions among subjects. We rejected, from all types of data, the trials in which the task had been incorrectly executed (e.g., movement execution during the observation phase) and those in which the hand had been incorrectly tracked by the Leap Motion Controller (e.g., swapping the thumb with the pinky finger). On average, we rejected thirty trials per subject from the total of 264. After rejection, each grasp type had a median of 7 trials in a 25% to 75% quartile range of 6.38 to 8 trials among all subjects. The movement onset was computed based on the accelerometer signal. Specifically, during the first second following the movement execution cue, we detected the first abrupt change in the signal’s mean and slope by using the Matlab function *findchanges*, which minimizes the sum of the residual (squared) error from the local mean.

Next, and only as a preliminary step to clean the EEG data of artifactual eye movements, sensor pops and electrocardiographic (ECG) contamination, we high-pass filtered the data above 1 Hz, using a Butterworth fourth-order, zero-phase filter. After aligning all the trials according to the movement onset, we extracted the first three seconds of the movement execution after the onset. We used the EEGLAB^59^ implementation of Semi-Automatic Selection of Independent Component Analysis (SASICA)^60^, which performs the correction of EEG data using several methods. For this analysis, we used ADJUST^61^ and the three EOG channels. After data cleaning, we projected the components into the channel space, maintaining the original frequency range between 0.1–40 Hz. We also used the extracted weights to clean the fixation period (1–3 s relative to the start of the trial). Next, we computed the time-frequency representation (TFR) using Morlet wavelets^62^ with a resolution of 0.5 Hz in the range 0.1–40 Hz for both the fixation period and the movement execution period. Finally, we calculated the event-related desynchronization (ERD/S) with respect to the baseline period, determined by the average of the two-second long fixation period of all the trials per subject. We calculated the ERD/S using the following formula from^63^:

$$ERD/S=\frac{{P}_{movement}-{P}_{baseline}}{{P}_{baseline}}\times \mathrm{100}{ \% },$$

where ${P}_{movement}$ is the power of the EEG signals during the movement execution in different frequency bands, and $\,{P}_{baseline}\,\,$is the subject-specific average power of the EEG signals during the fixation period, for the same frequency bands.

The eight EMG data channels were processed using Hilbert transform, standardized using *z*-score and, finally, the envelope (power) of the data was computed. From the signals recorded using the Leap Motion controller, we used the nineteen joint angles for the rest of the analysis. These joint angles correspond to the five proximal (one artificially created for the thumb and four for the other fingers), five intermediate and five distal joints of each finger, as well as the four in-between fingers joints. Given the high degree of correlation between some of the explored joints, we applied principal component analysis (PCA) to reduce the dimensionality of the kinematic data. We retained for each subject the first five components, which accounted on average for more than 95% of the data variance.

We assessed the within grasping condition variability between the grasping patterns of the three acquisition modalities, both at a repetition level and at a subject level, as described in^64^. We found a small within condition and within subject variability at a repetition level and a slightly larger inter-subject variability for all the recording modalities. We include the results of these analyses in the Supplementary Materials Figs S1 and S2.

### Feature extraction

Henceforth, for each of the three acquisition modalities (EEG, EMG and joint angles), we averaged single repetitions of the same grasp condition both within subject and between subjects leading to one group-level representation per grasp condition. For the averaging of the joint angles we used the circular mean, as implemented in the CircStat toolbox^65^.

For the extraction of the features, we defined three temporal windows of interest in the movement execution segment. The windows were consecutive and each window was 500 ms long, which is in line with the latencies of the grasping phases previously reported in the literature^66^. The first window was associated with the reaching and pre-shaping of the hand, and it started from the movement onset. The second window was associated with the finalization of the grasping movement. The third window contained information about the holding position of the grasping. From the three windows we extracted the patterns of interest from the three data acquisition modalities: EEG, EMG and joint angles.

For the EEG pattern extraction, we implemented a searchlight technique^67^ to extract ERD/S patterns simultaneously at different spatial locations and frequency bands. We present the searchlight approach in more detail in the Supplementary Materials Fig. S3. For the analysis, we defined 31 centroids in the channel space and 26 in the frequency space. One neighborhood, in either of the spaces, had five members: one centroid and four equidistant neighbors, and had 2 members in common with another neighbourhood. The two-dimensional window defined by the channel and frequency neighborhoods was slided across the two dimensions. Each frequency neighborhood corresponded to a 2 Hz-wide band and had an overlapping step of 0.5 Hz.

We applied the searchlight technique on each of the three windows of 500 ms from the movement execution segment. All the information within a two-dimensional window was concatenated into a vector. This vector defined the pattern of activity for the region with given centroid in space and in frequency. For the EMG envelope and for the kinematic joint angles, the behavioral patterns resulted from the concatenation of all channels (components) of data over each of the three inspected temporal windows.

### Representational similarity analysis

For each of the three temporal windows and acquisition modalities (EEG, EMG and joint angles), we compared the patterns of activity of the different grasping conditions, extracted previously. We used 1-*r* (where *r* is the Pearson correlation) as the distance metric between the patterns of different conditions. Next, we ranked and scaled the distances between 0 and 1, and computed the representational dissimilarity matrix (RDM)^52^ between pairs of grasping conditions. We present a conceptual description for the RDM calculation based on the grasp patterns extracted from our three acquisition modalities (EEG, EMG and joint angles) in the Supplementary Materials Fig. S4.

**Figure 2:**
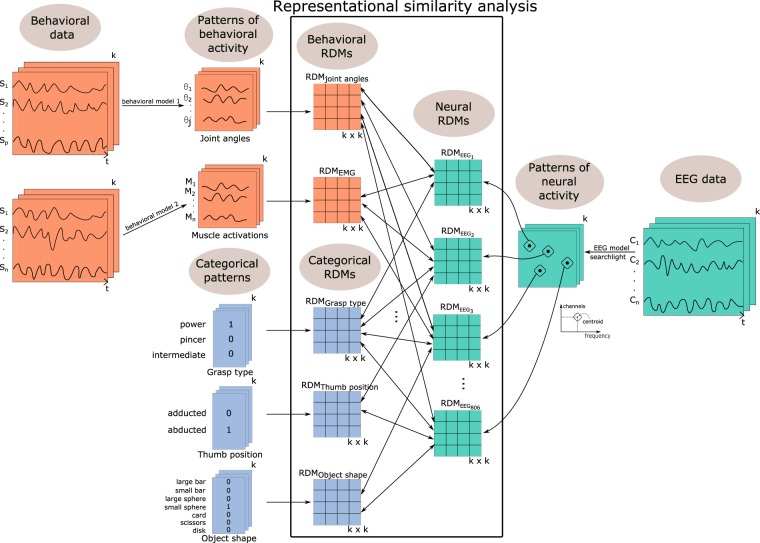
Representational similarity analysis (RSA) pipeline, including EEG searchlight analysis, computation of behavioral patterns, representation of categorical patterns and RDM computation for the three different modalities: EEG data (in green), behavioral data (in orange) and categorical models (in blue). As notations, we used *t* to indicate time, *k* for the 33 conditions, *s*_1_*..s*_*n(p)*_ indicate the sensors used to record behavioral data (*n* = 8 for muscle activity and *p* = 5 for the PCs of kinematic activity), *C*_1_*..C*_*m*_ the 64 channels for neural data recording, *M*_*1*_*..M*_*n*_ denote the muscle activations, while *θ*_*1*_..*θ*_*j*_ indicate the kinematic synergies derived from joint angles.

In addition to the brain and behavioral representations, we also implemented categorical models, as shown in Fig. 2. These models were built from previous categorizations based on the type of grasp, position of the thumb relative to the palm, defined in the largest pre-existing taxonomy of static and stable grasps^56^, and on the shape and size of the object.

The RDMs of these categorical models were binary representations, which contained a 0 for each pair of stimuli falling into the same category and a 1 for each pair of stimuli falling into different categories. We chose three categorical models: Grasp type, Thumb position and Object shape, which describe the relation between grasps based on intrinsic characteristics of groups of grasps (see Supplementary Materials Appendix B). The categorical model Grasp type described the relation between grasps based on a categorization in three types: power, precision and intermediate. The categorical model Thumb position showed the relation between grasps based on the position of the thumb: abducted or adducted. The categorical model Object shape depicted the relation between grasps based on the shape and size of the grasped object: large bar, small bar, large sphere, small sphere, disk, card and scissors. In an RDM, the representation of each grasp condition in the brain region of interest (or in the behavioral or categorical models) was compared to the representation of all the other conditions. Thus, each RDM leads to a representation of the overall differences between conditions that are encoded in that region of interest. Statistical inference determines which candidate (behavioral or categorical) model best resembles the reference (EEG) RDM in a specific brain region of interest.

In Fig. 2, we show the pipeline that we used to conduct representational similarity analysis (RSA)^52^. We used RSA in order to explore the similarities at a representational level between three types of data: EEG data (in green), behavioral data (in orange) and categorical data (in blue). From the preprocessed EEG data, we extracted the patterns of interest, by performing a searchlight analysis as presented previously and in more detail in Supplementary Materials Fig. S3. From the preprocessed behavioral data, we extracted patterns as transformations into kinematic synergies derived from joint angles and the envelope of muscle activations derived from electromyographic data, respectively, for the two types of behavioral data. For the categorical models, the patterns were much simpler, indicating the category to which each pattern belonged. As previously described, after extracting the patterns of interest from the individual subjects, we averaged these patterns to obtain condition-representative patterns at the group level.

In the center of Fig. 2, we emphasize the core of RSA within the large black rectangle. More precisely for each time window, we computed the RDMs for each of the three types of data by calculating the distances between the representations of each grasping condition. In the next step, we compared all the neural RDMs with all the behavioral and categorical RDMs. We used the 1-Spearman correlation to compute the distance between the ranked and scaled RDMs of the different modalities. For data visualization of the RDMs and RSA results we used the CET Perceptual color maps^68^.

To evaluate the consistency of our observed RSA effects we conducted a bootstrap analysis with 500 iterations. We performed the bootstrap analysis on a pool of 31 different samples corresponding to our subject number, and in each iteration we replaced the 31 samples with other randomly pulled 31 samples. In this manner, through bootstrapping we could estimate the confidence interval, as the variance of the mean (described by the distance between the reference and candidate RDMs) in our population. We report the 95% confidence interval (CI) of the procedure together with the observed mean effect. Hence, the population mean would be included within the ranges of 95% of the CIs of the mean calculated from repeatedly sampled data with a 95% probability.

To visualize the distances between the representations of different grasping movements, we used multidimensional scaling (MDS)^69^. MDS is a general dimensionality reduction method that projects entities in a low-dimensional space, such that their distances reflect their similarities. Specifically, similar entries will be located closer to one another, while dissimilar ones will be farther apart. For MDS visualization as a 2D plot, we used the Matlab function *mdscale*, which performs nonmetric MDS for two dimensions with the squared stress criterion.

Next, we performed a second-level analysis between all the representational models, in each of the three time windows. More precisely, we explored the similarities between RDMs in specific regions of interest (covering motor areas from central and parietal regions in mu and beta movement related frequency bands), behavioral RDMs and categorical RDMs. We again used MDS as an exploratory visualization technique to simultaneously relate all RDMs (from brain regions, behavioral representations and categorical models) to each other. The conciseness of the MDS visualization comes at a cost: the distances between entities are distorted (depending on the number of representations included); however, these distortions are accounted for by the thickness of the line connecting two entities. In a similar way to a rubber band, the connecting line becomes thinner when stretched beyond the length that would reflect the dissimilarity value that it represents, and thicker when compressed. Hence, the dissimilarity is a combination between the distance and the thickness of the connecting line. Nevertheless, this exploratory visualization technique provides a useful overall picture. It can alert us to relationships we had not previously considered and prompt confirmatory follow-up analyses.

## Results

Based on the assessment of the Edinburgh Handedness Inventory included in the Supplementary Materials (Appendix A), we found that all the subjects were dominantly right-handed.

### Reference and candidate representations

We computed the reference representations based on the searchlight analysis of the time-frequency representation of the EEG data in the three time windows of interest: hand pre-shaping, reaching of the final grasping posture and holding. The searchlight analysis yielded a total of 806 (31 space centroids × 26 frequency centroids) reference RDMs for each window. The candidate representations are based on the behavioral patterns (envelope of the EMG activations and joint angles kinematics). In addition, we also used as candidate representations three categorical models, based on the type of grasp, the position of the thumb relative to the palm and the shape and size of the object. We present in the Supplementary Materials Fig. S5 the candidate RDMs for the behavioral representations and for the categorical models.

For the behavioral representations, we observed that the differences between the grasps were preserved over the course of the three windows, with some small changes. The first window, corresponding to the hand-preshaping phase was slightly more different than the other two windows. Specifically, for the EMG RDMs the distance between the first window and the second was 0.1, and 0.16 relative to the third. For the kinematic RDMs the distance between the first window and the second was 0.24, and 0.33 relative to the third. The second and the third windows were highly similar for both muscle and kinematic modalities, with a distance of 0.04. Furthermore, we also found high similarities between the two behavioral models, which are in line with previous findings^70^. We observed clusters of grasp conditions involving elongated or spherical objects.

### Similarity between the candidate representations and the reference representations

**Figure 3:**
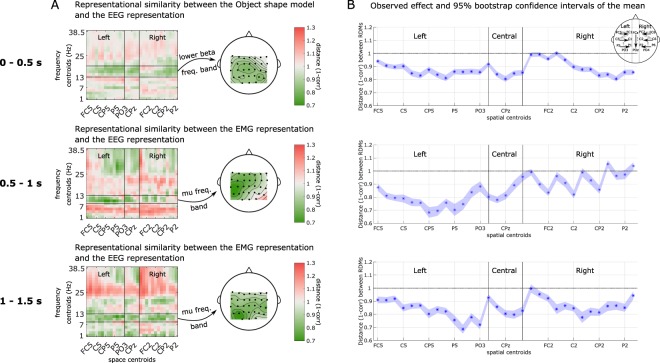
(**A**) Representational similarity analysis between the candidate representations and the reference representations extracted through a searchlight implementation. The vertical black lines mark the area covered by the midline centroids. The horizontal lines mark the frequency band written on the arrows. The space centroids are sorted from periphery-to-midline and anterior-to-posterior. Topographical representation of the similarity in the specified frequency bands. On the first row, the dissimilarity w.r.t. the categorical Object shape model in the time window between 0–0.5 s. The second and third rows show the dissimilarity w.r.t. the EMG representation in the time window 0.5–1 s and 1–1.5 s. (**B**) Bootstrapping results of the RSA for the three time windows. The contralateral and central brain regions show smaller distances (higher correlations) between the reference (EEG) representation and the candidate (categorical Object shape and behavioral EMG) representations in the centro-parietal and parietal regions. The shaded area indicates the 95% confidence interval computed after 500 bootstrapping iterations.

Next, we present the exploratory results of the representational similarity (RSA) analysis between the EEG representations extracted through the searchlight analysis, and the behavioral and categorical representations, for the three time windows. Specifically, we investigated the relationship between the candidate RDMs and the reference EEG RDMs. A complete overview of the exploratory results for all the comparisons between the reference RDMs and the candidate RDMs can be found in the Supplementary Materials Fig. S6. We chose to focus on the similarities observed in the frequency bands known a priori to contain motor-related information, such as the mu and beta bands. Hence, panel A of Fig. 3 presents the topoplot of the similarity representation in areas that showed in Fig. S6 the largest similarity between behavioral representations or categorical models and EEG reference representations for each of the three time windows. We observe that the centro-parietal regions in the lower beta frequency band had the most similar RDMs with the Object shape categorical model in the hand pre-shaping movement phase (0–0.5 s). In the grasp finalization movement phase (0.5–1 s) and in the holding phase (1–1.5 s), we found a higher degree of similarity between the left centro-parietal EEG RDM and the EMG RDM in the mu frequency band.

The regions with the smallest distance between RDMs are the left centro-parietal and parietal in the mu and lower beta frequency bands. In the first time window, the observed effects of dissimilarities noted between the reference EEG RDMs and the Object shape model in the lower beta band were lower in the contralateral centro-parietal areas as well as in the ipsilateral parietal areas. We observed a dissimilarity effect of 0.8 with a 95% CI: [0.78, 0.82]. In the second time window, we found dissimilarities between the reference EEG RDMs and the EMG RDM in the mu band, which were lower in the contralateral centro-parietal areas. We observed a dissimilarity effect of 0.68 with a 95% CI: [0.64, 0.72]. In the third time window, we observed dissimilarities between the reference EEG RDMs and the EMG RDM in the mu band, which were lower in the contralateral parietal areas. We observed a dissimilarity effect of 0.68 with a 95% CI: [0.65, 0.72]. In panel B, we show the observed effect of similarity over all the centroids, as depicted in panel A, together with the 95% confidence interval of the procedure computed after 500 bootstrapping iterations.

**Figure 4:**
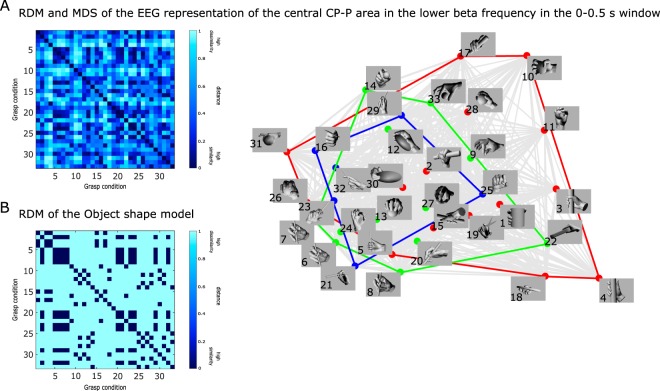
Relation between grasps during hand pre-shaping phase based on: (**A**) the RDM and MDS of the EEG representation of the centro-parietal and parietal (CP-P) brain regions in the lower beta frequency band, and (**B**) the RDM of the categorical Object shape model. The colors of the MDS correspond to the more conventional type categorization of grasps: red indicates power grasps, green, precision grasps and blue, intermediate grasps. The grey color indicates all the pairwise relations between grasps.

For an intuitive visualization of the relationship between the different grasping conditions, we used multidimensional scaling (MDS). Panel A of Fig. 4 illustrates the relation between grasp conditions as encoded in the hand pre-shaping phase (0–0.5 s after movement onset) by the centro-parietal and parietal regions. On the left side, we present the RDMs among grasping conditions for the EEG and for the categorical Object shape model. On the right side we show the MDS representation, based on the differences in brain patterns.

On closer inspection, we found that the grasps that involved objects with a thinner shape were clustered together (see the lower left part of the MDS representation). Surprisingly, we found that the categorization into grasp types—power, pincer and intermediate—depicted by the three colors in the MDS plot, was not congruent with the reference EEG RDM representation. Panel B shows the relation between grasp conditions based on the shape categorization. Specifically, the categorical Object shape RDM contained a 0 for each pair of grasps that fell into the same category and a 1 for each pair of grasps that fell into different categories. We found a correlation of 0.2 between the neural RDM and the categorical Object shape model during the first movement phase.

**Figure 5:**
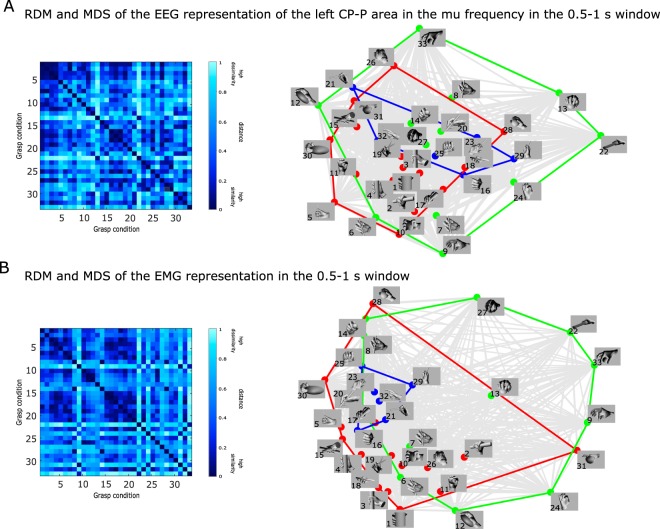
Relation between grasps during the grasp finalization phase based on: (**A**) the RDM and MDS of the EEG representation of the left centro-parietal and parietal (CP-P) brain regions in the mu frequency band, and (**B**) the RDM and MDS of the behavioral EMG representation. The colors of the MDS correspond to the more conventional type categorization of grasps: red indicates power grasps, green, precision grasps and blue, intermediate grasps. The grey color indicates all the pairwise relations between grasps.

In Fig. 5, we show the distance between the grasp representations in the time window associated with the grasp finalization (0.5–1 s), as encoded by EEG neural patterns and by the behavioral EMG patterns.

In panel A, we present the RDM and the MDS of the relation between the grasp conditions as covered by the left centro-parietal region of the brain in the mu frequency band. Panel B depicts, through the RDM and MDS, the relation between the grasp conditions as encoded by the EMG patterns.

We used MDS to visualize the similarity structure of each RDM. This exploratory visualization technique (Fig. 5A,B right) allowed us to simultaneously relate all grasping conditions to each other. It, thus, provides a summary of the information we would obtain by inspecting the RDM representation (shown in Fig. 5A,B left). The grasps that involved elongated objects were clustered together in both EEG and EMG representations. For example, the first 7 grasps get grouped into similar clusters in both types of representations. Also, grasps 22, 24, 9, 13, 33 form a distinct cluster that is further apart from most of the other grasps (pointing towards larger dissimilarities between the grasps located within this cluster and the rest of the grasps) in both types of representations.

**Figure 6:**
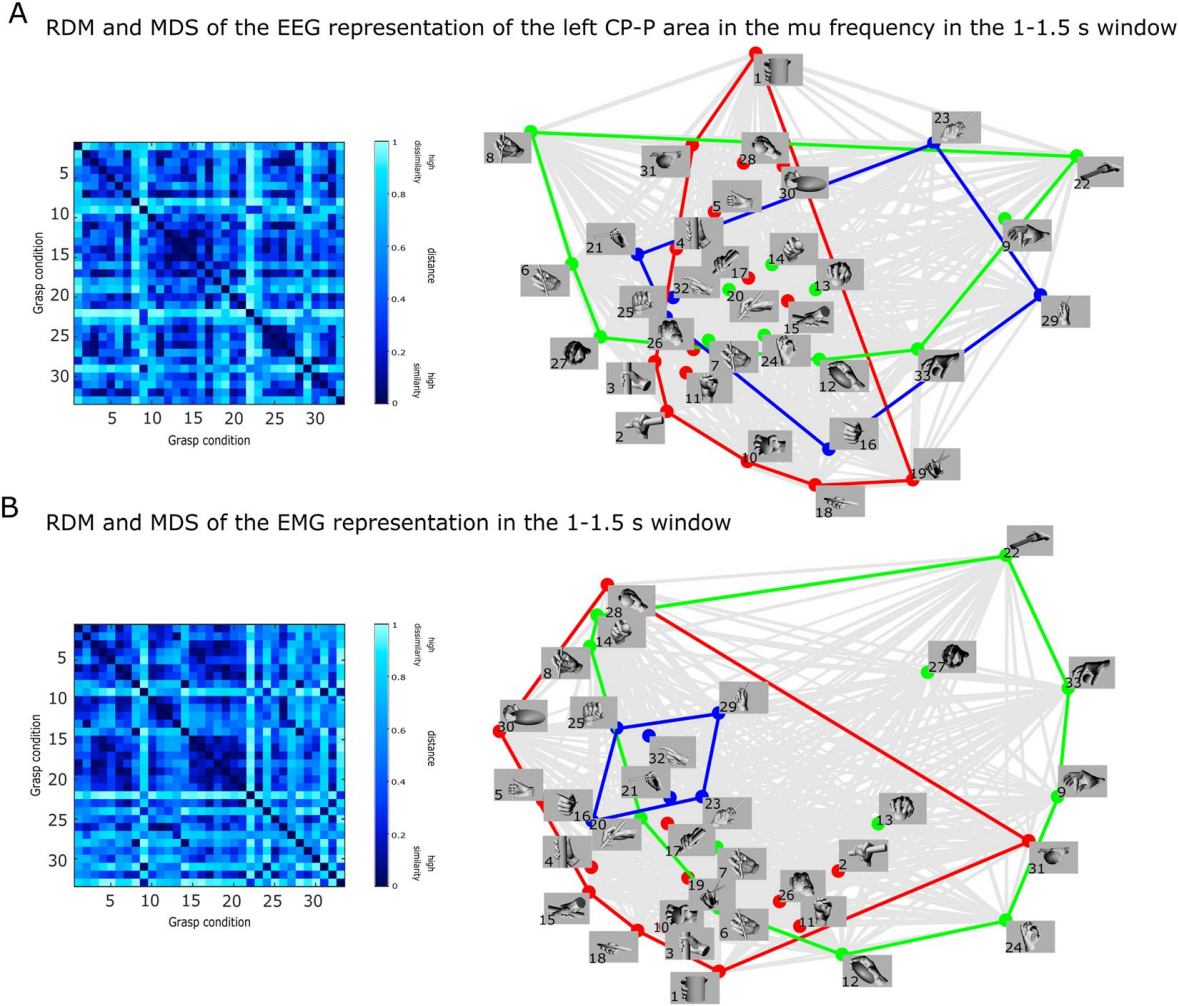
Relation between grasps during the holding phase based on: (**A**) the RDM and MDS of the EEG representation of the left centro-parietal and parietal (CP-P) brain regions in the mu frequency band, and (**B**) the EMG RDM behavioral representation. The colors of the MDS correspond to the more conventional type categorization of grasps: red indicates power grasps, green, precision grasps and blue, intermediate grasps. The grey color indicates all the pairwise relations between grasps.

In Fig. 6, we show the relation between grasps in the time window associated with the holding of the final grasp posture (1–1.5 s), as encoded by EEG neural patterns and the behavioral EMG patterns. In panel A, we present the RDM and the MDS of the relation between the grasp conditions as covered by the left centro-parietal region of the brain in the mu frequency band. Through the RDM and MDS, Panel B depicts the relation between the grasp conditions as encoded by the EMG patterns during the holding phase of the grasping movement.

In this phase of the grasping movement, we observed that the EEG pairwise representation between grasps changed relative to those seen in the previous window, in which the grasping movement was still ongoing. On the other hand, the EMG representation remained stable compared to the one observed in the previous window. However, using the MDS visualization, we found that several grasps maintained their relative distances between representational types. As we had previously observed, the grasps that involved elongated objects clustered together also in the holding phase. We also found that some grasps, such as 9, 22, and 29, have relatively large distances relative to the majority of grasps. This relation between groups of grasps can be observed both in the EEG and in the EMG representational modalities.

### Similarity analysis between representational models

**Figure 7:**
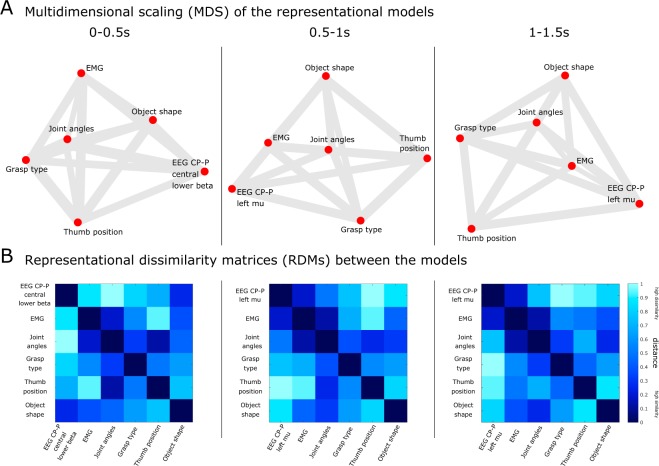
Relations among the reference (EEG-driven) and candidate representations (established by EMG, joint angles, or by categorical descriptors, such as grasp type, thumb position and shape of the object) in three 500-ms time long windows after the movement onset. (**A**) Multidimensional scaling of the (dis)similarity among the representational models. (**B**) Second level RDMs among representational models.

Next, we conducted a second-level analysis to explore all the pairwise relations between the reference and candidate representations. Fig. 7 shows the pairwise relations between the EEG representations of grasping from the CP-P (centro-parietal and parietal) regions and other candidate representations: EMG, joint angles and three categorical models (i.e., Grasp type, Thumb position and Object shape) over the course of three grasping stages: hand pre-shaping (0–0.5 s), attainment of the final grasping posture (0.5–1 s) and holding the grasping posture (1–1.5 s).

In Fig. 7, we used MDS (Fig. 7A) to visualize the similarities among the structure of activity patterns in the centro-parietal areas and behavioral and categorical representations. Here, we chose to use MDS to visualize the similarity structure among all the RDMs. We assembled all the pairwise comparisons between the RDMs of the reference and candidate representations in a dissimilarity matrix of RDMs (Fig. 7B), using rank-correlation as the dissimilarity measure. We then performed MDS on the basis of this second-order dissimilarity matrix. The use of this exploratory visualization technique allowed us to simultaneously relate all RDMs (from categorical models, behavioral and brain representations) to each other. The dissimilarity between pairs is expressed by both the distance and the thickness of the connection. Due to higher dimensionality of the pairwise comparisons, the distances are distorted, and there are no error bars or statistical indicators. Nevertheless, this overview visualization revealed relations we had not previously considered and could prompt confirmatory follow-up analyses.

Panel A shows a smaller distance between the EEG representation and the Object shape model in the hand pre-shaping phase. In the next two grasping phases, the EEG representation encoded the EMG representation to a higher extent with a distance of 0.68 (which is a moderate correlation of 0.32), as shown in Panel B. Moreover, we also observe in Panel B a small distance of 0.36 (high similarity, *r = *0.64) between the EMG and the kinematic representation based on the joint angles.

## Discussion

In the current study, we explored the similarities among the EEG neural patterns (using a searchlight approach), behavioral patterns and categorical models of the largest repertoire of unique grasping movements provided in literature^56^. We found that the parietal and centro-parietal motor areas encode activity about grasping movements. More specifically, we found that—during the reaching and pre-shaping phases of the movement—the model that categorizes grasps based on the shape and size of the virtually grasped objects best describes the EEG representation from the centro-parietal and parietal regions in the lower beta frequency band. During the next grasping phases, in which the final grasping position is attained and held, we found moderate similarities among the EEG representations from the centro-parietal and parietal areas in the mu band and the EMG representations. Interestingly, the kinematic representations based on joint angles were highly similar to the EMG representations; however, the similarity between the former and the EEG reference representations was lower, indicating that the kinematic representations may contain additional grasping information that is not encoded in the EEG brain patterns.

In the brain-computer interface (BCI) research, there has been a strong endeavor to decode reliable control signals from the brain, with the aim to control computer cursors, neuroprosthetics, or robotic limbs. Given the engineering and translational goals of BCI research, we often disregard the neural plausibility of the decoding results. Consequently, the decoding results should not be interpreted as a neural representation of the way in which the brain processes a certain task. This incorrect interpretation of decoding findings has been widely disseminated, and it is also known as the “Decoder’s Dictum”^30^. This dictum states that “if information can be decoded from patterns of neural activity, then this provides strong evidence about what information those patterns represent”. A good argument for the reasons of why the dictum is false has been given by Ritchie and colleagues in^30^. We believe that, complementary to the state-of-the art decoding findings, we should try to clarify our understanding of the decoding results by involving inferences about neural functions and their representations regarding their behavioral resemblances^71^. One way to make better inferences is by leveraging the benefits of exploratory analysis, which has the potential to address broader scientific questions and inform about promising effects observed in the data^50,51^. New BCIs could be developed based on the unitary understanding of neural functions from different brain areas and the associated behavior. For instance, Aflalo and colleagues^72,73^ have recently suggested that signals from parietal cortex rather than motor cortex can be used to control the reach-and-grasp movement of a neuroprosthetic arm in a patient with spinal cord injury.

In this study, we addressed three questions: (1) Can EEG signals capture the differences between multiple types of grasps; (2) Which grasping covariate best explains how grasps are represented at the EEG level; (3) To what extent do grasping representations based on movement covariates and on EEG patterns resemble each other. To address these questions, we designed a paradigm that incorporates the execution of a large number of distinct grasping movements while simultaneously recording neural, muscle and kinematic correlates of the grasps. To explore the relations among the neural, behavioral and categorical patterns of different grasping movements, we chose to conduct a representational similarity analysis (RSA). Because it is a multivariate pattern analysis method, the use of RSA allowed us to study both the representational geometries and informational content of the grasp conditions in multidimensional space spanned by EEG sensors, different frequency bands and time points. This characteristic makes RSA particularly amenable for searchlight analysis. Moreover, RSA is especially suitable for use with condition-rich designs such as ours (33 different grasp movements). As with classifier decoding, RSA is a technique that is sensitive to information encoded in patterns of activity. However, rather than attempting to determine what information can be (linearly) read from the patterns of activity, the use of RSA allowed us to characterize the representational geometry of a rich set of conditions and compare it to various representations or models. Moreover, rather than allowing us to compare, for instance, brain and muscle or kinematic patterns, which requires defining correspondence mapping between pattern units and channels of activity), the use of RSA allowed us to compare the representations at the level of dissimilarity matrices (between conditions) of each model^30,53^.

In two recent studies, RSA has been applied either to study the encoding properties of models based on muscle and kinematic synergies in different cortical areas during grasping movements^26^, or to characterize the relation between behavioral and neural information based on the comparison of dissimilarity matrices in the case of single or multi-finger movements^28^. In these studies the description of the behavioral models was based either on the movement synergies or on the information extracted from the activity of the whole postural vector. Recently, Leo *et al*.^26^ conducted a study combining kinematic, electromyographic and fMRI brain activity and found that the hand postural information encoded through kinematic synergies during grasping is represented in cortical areas related to hand movement control. In our exploratory study, we found the activation of the same brain areas. However, we observed larger similarities between the EEG and the muscle representations than relative to the kinematic representation. A comparison between the performance of the behavioral models (muscle and kinematic) in our study and the ones presented in^26,28^ is not straightforward because we recorded the muscle activity from eight extrinsic (located on the forearm) hand muscles, while the previous studies recorded from both intrinsic (located on the hand) and extrinsic hand muscles, with a larger number of sensors on the intrinsic muscles. The extrinsic muscles of the hand are larger than the intrinsic ones, thus, the recorded signals are often collinear leading to a higher amplitude and a better signal-to-noise ratio when compared to the intrinsic muscles of the hand. The good performance of our muscle model could be attributed to the quality of the signals recorded from the extrinsic muscles. Also, the kinematic models are differently obtained in the current study than in previous ones (different acquisition systems, number of components extracted, etc.), making a direct comparison more difficult. Furthermore, we believe that one advantage in our study is the simultaneous acquisition of all three recording modalities (EEG, EMG and kinematics). In previous studies each modality was acquired separately, this could lead to differences in the recordings due to the nonstationarity of the signals or the state of the subjects across the sessions. The synchronous acquisition of all three modalities can allow a potentially better characterization of the grasping movements from different perspectives.

From a behavioral perspective, we can grasp an object in many different ways. The goal of the movement and the visual properties of the object play important roles in the pre-shaping of the hand into an appropriate configuration. For instance, grasping a knife to cut bread is different than grasping a knife to perform surgery. Fabbri *et al*.^27^ explored the relation between grasp and object properties, such as shape, size and elongation, in terms of their representation in fMRI activity. They observed that object elongation is the most strongly encoded object feature during grasping. Moreover, they found that the number of fingers used during grasping is represented both within ventral-stream and parietal regions. In our study, we found activations in the parietal and centro-parietal areas during the hand pre-shaping phase that resemble the categorization of grasps based on the shape property of the objects. Moreover, we also found that the elongation of the objects is a distinctive feature that structures different grasping movements into clusters. Yokoi *et al*.^29^ investigated the representation of sequences of finger movements in the motor cortex. Although it is well-known that the human primary cortex (M1) is the main region involved in single finger movements, it is debatable if sequences of finger movements also originate in the M1 or involve other areas. The reliable differences between different sequences of finger movements seem to be encoded in premotor and parietal regions^29^. Our results are in line with these findings, supporting the activation of parietal and centro-parietal areas during grasping, as a meaningful sequence of finger movements.

One potential limitation of the current study is the absence of real objects during grasping. We chose to use imaginary objects in order to remove potential confounds driven by force or feedback differences between the grasped objects, which could contaminate the recorded signals. In studies that have explored the differences between grasping movements towards real and imaginary objects^74–76^, the first few components, after dimensionality reduction methods had been applied, exhibited high degrees of similarity between the two types of grasping. Furthermore, in the current setup, real objects could occlude the fingers and lead to errors in the tracking signals. In terms of ecological validity, we aimed to increase the consistency in the execution of each movement for each subject. Given the small number of repetitions per grasping movement, we instructed each subject to perform all the repetitions of the same movement in a consist manner. This movement constraint can hinder the ecological validity of the task; however, we did not constrain the inter-subject variability. Moreover, we found that although the subjects showed similar behavioral patterns among the repetitions of the same grasping movement, their neural correlates were more variable. Potential reasons for this variability observed in the neural patterns can be attributed to the lack of consistency in the mental strategy or to a decrease in the level of engagement of some subjects while performing repeated movements of the same grasp. The movements were performed on a natural pace and none of them was unfamiliar or difficult to perform for the subjects. From an experimental design perspective, we made a trade-off between each condition’s specificity (which would require the acquisition of more repetitions of the same grasp type from each subject) and among-conditions sensitivity (which allowed us to capture the structure of the differences among many grasping conditions). Thus, this design is not suitable for decoding studies, which do not require so many different conditions, but a better characterization of each condition.

In summary, these findings allow us to frame hypotheses regarding the representation of distinct grasping movements based on the relation between their EEG representations and the associated behavioral (muscle or joint angles) representations, as well as relative to categorical models, describing the object’s size and shape, type of grasp, or position of the thumb relative to the palm. On a group-level, we found that the EEG representation of grasping finalization and holding the final grasping position is encoded to a higher extent in the muscle representations than in the kinematic representations. However, the two behavioral representations were highly correlated. By conducting a follow-up confirmatory analysis in a future study, we will be able to evaluate the encoding of the mixed behavioral representation in the neural patterns. Furthermore, we plan to test the proposed categorical Object shape model and confirm whether it is a suitable model for the representation of grasps during the hand pre-shaping phase. We encourage other researchers in the BCI field to explore and evaluate also other behavioral and categorical models than the ones presented here, or a combination of those.

We strongly believe that a good understanding of neural mechanisms can be inferred only by incorporating the behavioral covariates. Such inferences, once collected, could allow us to gain more natural control of robotic devices or neuroprostheses in the near future.

## Electronic supplementary material

Supplementary Materials

## Data Availability

Datasets and code available at: https://osf.io/my5pt.
