# Supplementary material for: Exploring representations of human grasping in neural, muscle and kinematic signals
Source: Sci Rep. 2018 Nov 12;8:16669. doi: 10.1038/s41598-018-35018-x (PMC6232146; doi:10.1038/s41598-018-35018-x)
Supplement: Supplementary file 1 — Supplementary Materials [file 41598_2018_35018_MOESM1_ESM.pdf]

Supplementary materials

Exploring representations of human grasping in neural, muscle and kinematic signals

Andreea I. Sburlea[1], Gernot R. Müller-Putz[1,*]

[1]Institute of Neural Engineering, Graz University of Technology, Austria

**corresponding author*

andreea.sburlea@tugraz.at, gernot.mueller@tugraz.at

Figures

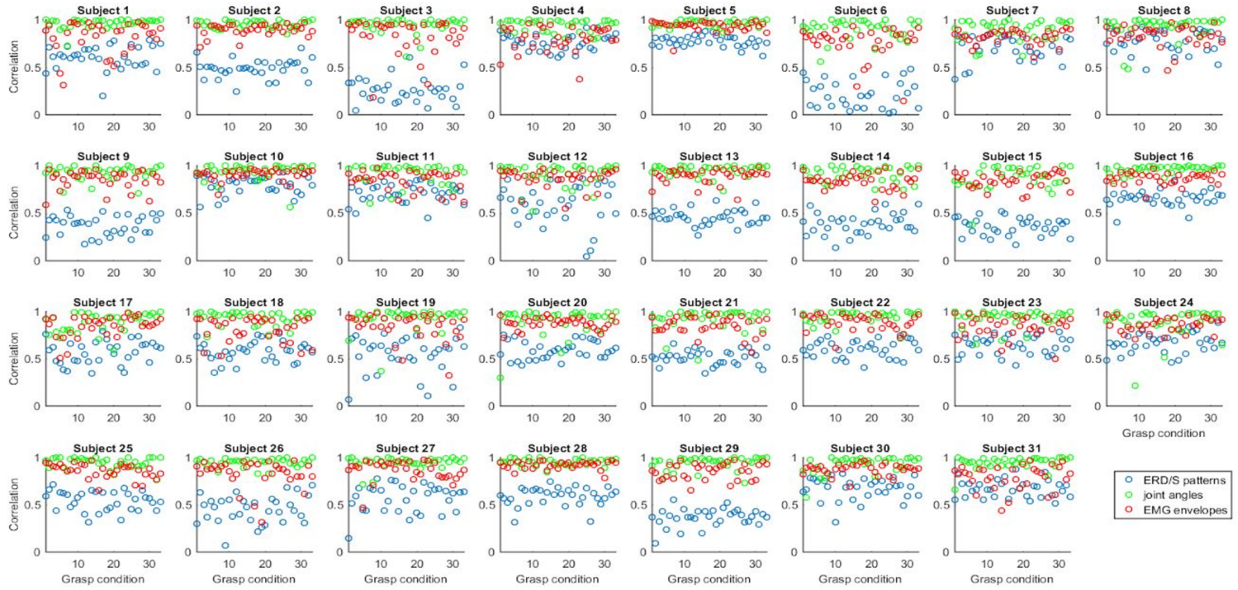

Figure S1. Split-half correlation values between the grasping repetitions. The colors indicate the patterns of the three investigated modalities. The patterns consisted in the ERD/S patterns from motor and parietal channels in the mu and beta band, first five PCs of the joint angles and the EMG envelopes. Each circle indicates the correlation of the repetitions within a grasping condition.

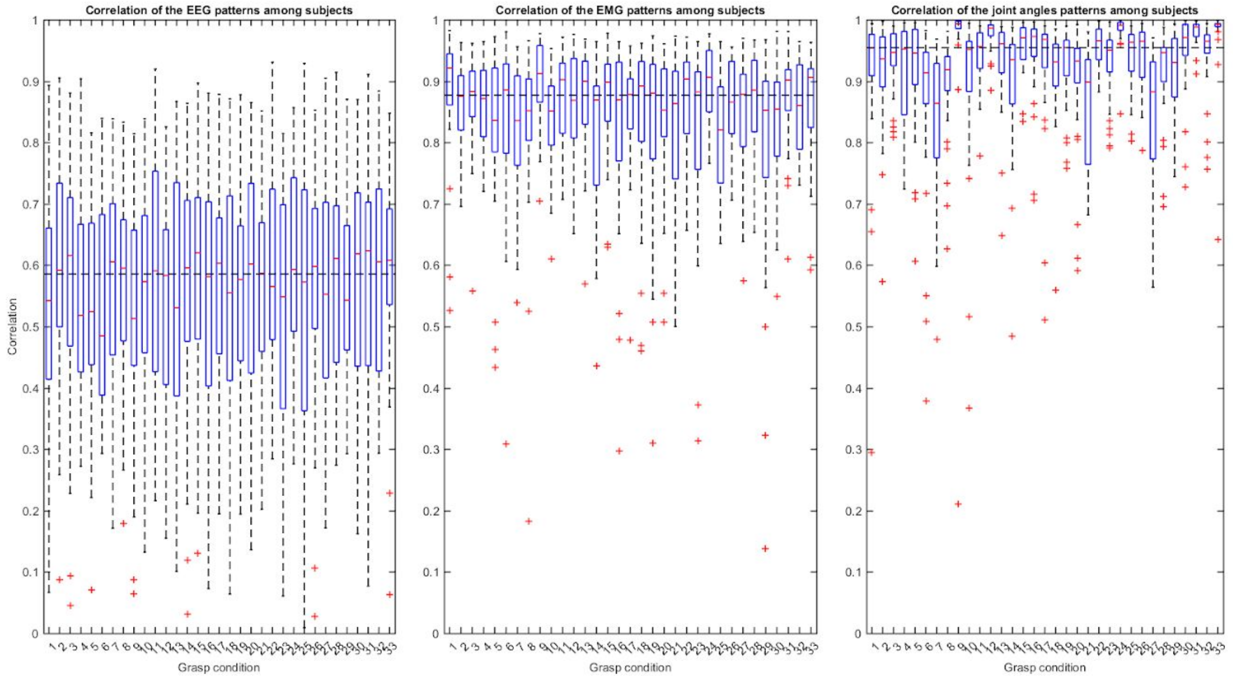

Figure S2. Summary statistics as measures of correlation among subjects for each grasp condition and recording modality. The first subplot shows the correlation between the EEG brain patterns of all subjects for each grasp condition. The second subplot shows the correlation between the EMG patterns of all subjects. The third subplot shows the correlation between the principle components of the joint angles of all subjects. The black dashed line indicates the overall median value of correlation across all grasps.

We observed that the behavioral patterns showed higher correlations than the brain related patterns. The summary statistics (mean $\pm$ std) for the correlations among subjects and grasps are: 0.56 ± 0.19 for the ERD/S patterns, 0.85 ± 0.11 for the EMG envelopes and 0.92 ± 0.1 for the joint angles. We observed that the brain related patterns showed large inter-subject variability than the behavioral patterns.

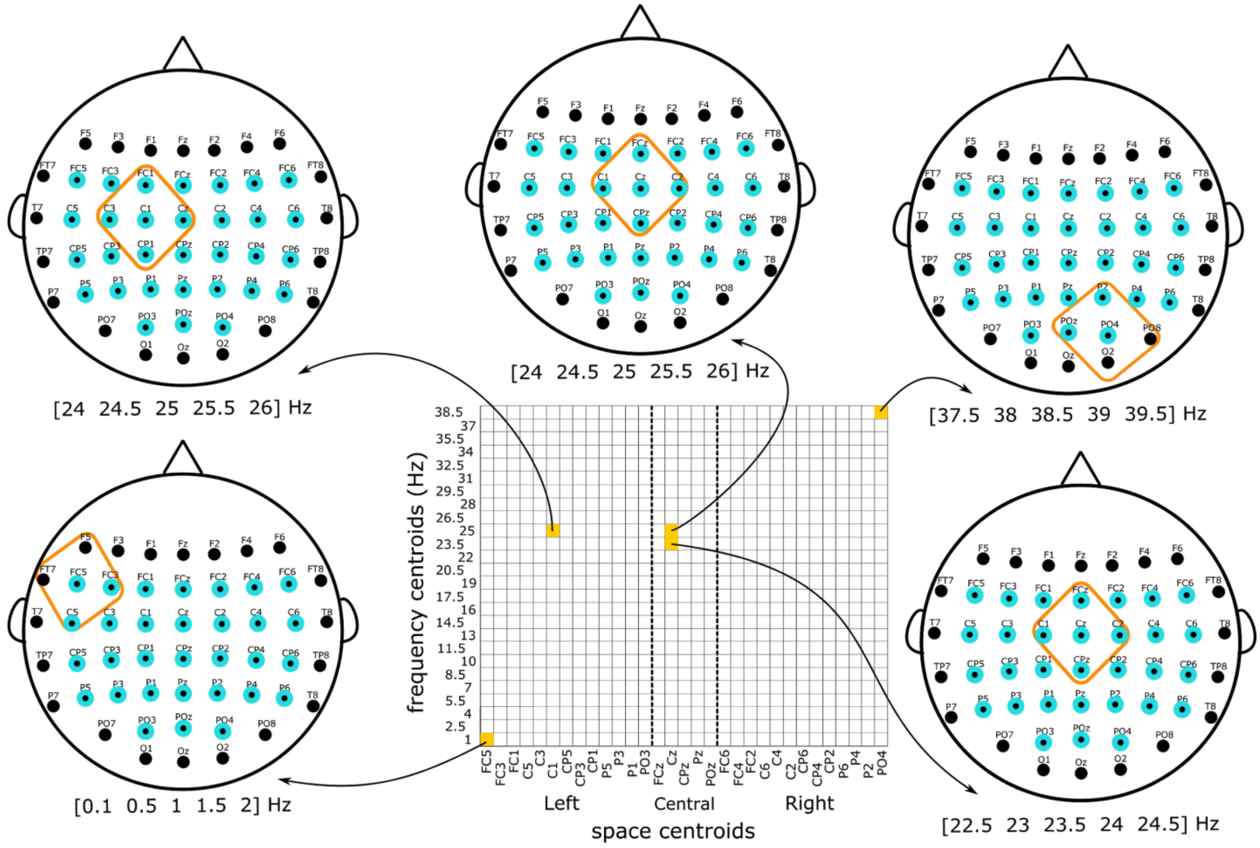

Figure S3. Searchlight analysis. Simultaneous extraction of patterns of activity of different grasps from EEG space and frequency neighborhoods determined by the associated centroids. The matrix in the middle shows all the covered combinations of centroids. As examples, we highlight the first and the last combination, as well as another three, out of which two are sharing either a common neighborhood in space or in frequency. The topoplots show the selected neighborhoods in space and in frequency. The blue circles in the topoplots mark the channels that were selected as centroids.

We implemented a searchlight technique [67] to extract ERD/S patterns simultaneously at different spatial locations and frequency bands. We defined 31 centroids in the channel space and 26 in the frequency space. One neighborhood, in either of the spaces, had five members: one centroid and four equidistant neighbors. The two-dimensional window defined by the channel and frequency neighborhoods was slided across the two dimensions. Examples of such two-dimensional windows are highlighted in yellow in central matrix from Figure S3. In the channel space, due to the distribution of the electrodes on the head, we excluded the peripheral channels with fewer neighbors. The space centroids, showed in light blue in Figure S3, are: FC5, FC3, FC1, C5, C3, C1, CP5, CP3, CP1, P5, P3, P1, PO3, FCz, Cz, CPz, Pz, POz, FC6, FC4, FC2, C6, C4, C2, CP6, CP4, CP2, P6, P4, P2, PO4. Each neighborhood has two members in common with another neighborhood. For example, if C3 is a centroid, it has FC3, C5, CP3 and C1 as neighbors. When slided and C1 becomes a centroid, C3 will be one of the neighbors. The frequency centroids ranged from 1 to 38.5. Each frequency neighborhood corresponded to a 2 Hz-wide band and had an overlapping step of 0.5 Hz.

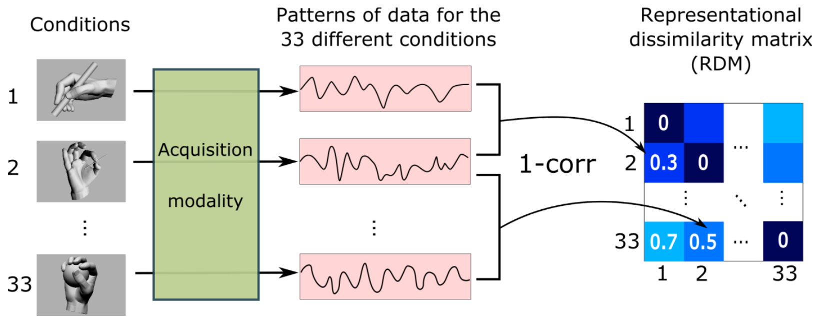

Figure S4. Representational dissimilarity matrix (RDM) calculation for a generic case of an acquisition modality, built on the comparison between the extracted patterns of each condition.

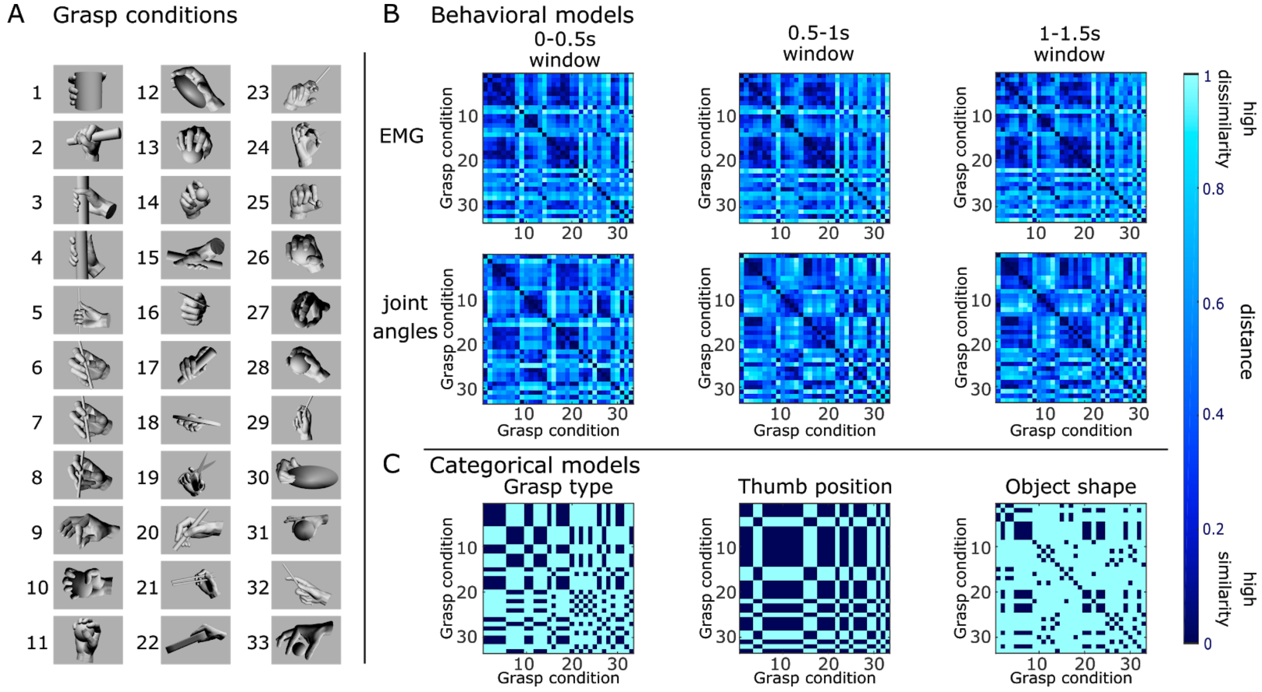

Figure S5. Representational dissimilarity matrices (RDMs) of the candidate representations. A. Pictograms of the grasping movements. B. The RDMs of the behavioral representations: EMG and joint angles, for the three windows: 0-0.5s, 0.5-1s and 1-1.5s. C. The RDMs of the categorical models: Type, Thumb and Shape. The linear color bar indicates ranked and scaled distance between 0 and 1, where 1 corresponds to a large distance between grasps and 0 to a smaller distance and, therefore, a higher similarity.

For the behavioral representations, we observed that the differences between the grasps were preserved over the course of the three windows. Specifically, we found that elongated objects, such as the ones in the first seven grasp conditions, were grouped together with other elongated objects, such as the ones between 15 and 21, as well as with 23, 29 and 32, in both behavioral representations. We also found clusters of spherical objects such as 11, 12, 13 and 26. The joint angles representation—in contrast to the EMG representation—also captures the similarities with the other spherical objects, such as 26, 27, 28, 31 and 33.

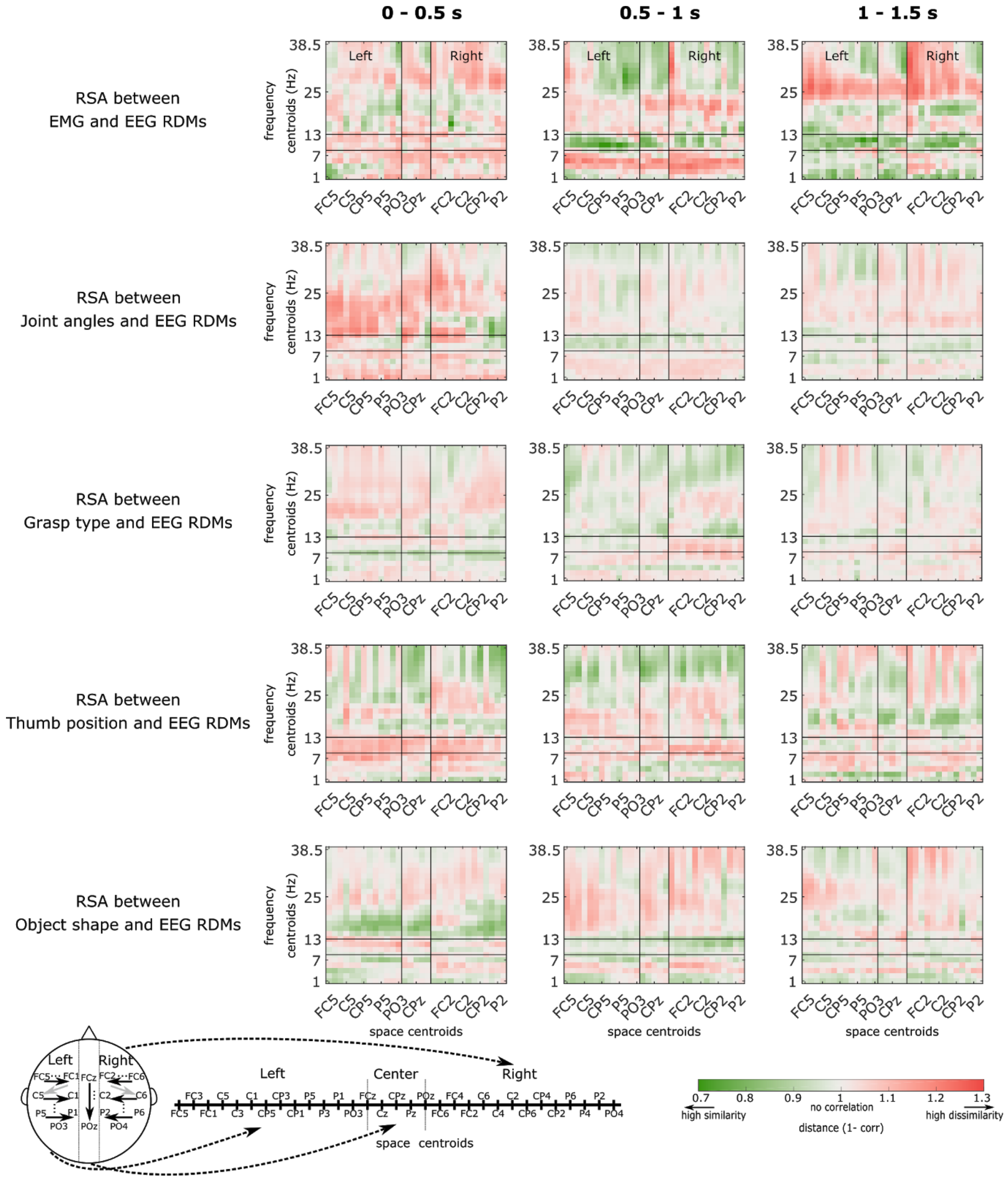

Figure S6. Representational similarity analysis between the candidate representations and the reference representations extracted through a searchlight implementation. The vertical black lines mark the area covered by the midline centroids. The horizontal lines mark the mu (8-13 Hz) frequency region. The ordering of the space centroids follows the description from the drawing next to the color bar. Specifically, centroids are sorted from periphery-to-midline and anterior-to-posterior.

In Figure S6, we show the exploratory results of the representational similarity (RSA) analysis between the EEG representations extracted through the searchlight analysis, and the behavioral and categorical representations, for the three time windows, ordered by columns. Specifically, we investigated the relationship between the candidate RDMs and the reference EEG RDMs. Each row of the figure shows the similarity patterns between one candidate representation and all the reference EEG RDMs.

In the first time window, which is associated with the reaching and hand pre-shaping movement, we observed that the categorical Object's shape model had the smallest dissimilarity (1-*r* = 0.8, SEM = 0.010) with the reference EEG representations from the lower beta band in the centro-parietal areas of both hemispheres. Also, in the higher frequencies (above 25 Hz), reference representations showed similarities with the categorical Thumb model in the central and ipsilateral areas of the centro-parietal region (1-*r* = 0.82, SEM = 0.010).

In the second time window, which is associated with the finalization of grasping movement, we observed the smallest dissimilarity (1-*r* = 0.68, SEM = 0.012) between the EMG and the EEG representations in the mu band in the centro-parietal area, which was mostly pronounced in the left hemisphere. We also found similarities in the lower gamma frequency, also located in left centro-parietal regions (1-*r* = 0.72, SEM = 0.013). The higher frequency reference representations also showed similarities with the categorical Thumb model (1-*r* = 0.78, SEM = 0.008) in the pre-central areas, and with the categorical Type model (1-*r* = 0.83, SEM = 0.009) in the ipsilateral parietal areas.

In the third window, which is associated with the holding phase, the EMG representation had the lowest dissimilarity (1-*r* = 0.68, SEM = 0.013) with the EEG representations from the mu band in the centro-parietal and parietal regions of the contralateral hemisphere, and a slightly greater dissimilarity in the right hemisphere in the same frequency range (1-*r* = 0.76, SEM = 0.015).

Appendix A. Edinburgh Handedness Inventory

Instructions
For each of the activities below, please indicate:
Which hand you prefer for that activity?
Do you ever use the other hand for the activity?

**Which hand do you prefer to use when:**		**no pref**			**Do you ever use the other hand?**
**Writing:**	Left		Right		Yes
**Drawing:**	Left		Right		Yes
**Throwing:**	Left		Right		Yes
**Using scissors:**	Left		Right		Yes
**Using a toothbrush:**	Left		Right		Yes
**Using a knife (without a fork):**	Left		Right		Yes
**Using a spoon:**	Left		Right		Yes
**Using a broom (upper hand):**	Left		Right		Yes
**Striking a match:**	Left		Right		Yes
**Opening a box (holding the lid):**	Left		Right		Yes
**items below are not on the standard inventory:**					
**Holding a computer mouse:**	Left		Right		Yes
**Using a key to unlock a door:**	Left		Right		Yes
**Holding a hammer:**	Left		Right		Yes
**Holding a brush or comb:**	Left		Right		Yes
**Holding a cup while drinking**	Left		Right		Yes

The evaluation criteria:

Laterality Index (LI)	Decile
LI = −100	10th left
−100 ≤ LI < −92	9th left
−92 ≤ LI < −90	8th left
−90 ≤ LI < −87	7th left
−87 ≤ LI < −83	6th left
−83 ≤ LI < −76	5th left
−76 ≤ LI < −66	4th left
−66 ≤ LI < −54	3rd left
−54 ≤ LI < −42	2nd left
−42 ≤ LI < −28	1st left
−28 ≤ LI < 48	Middle
48 ≤ LI < 60	1st right
60 ≤ LI < 68	2nd right
68 ≤ LI < 74	3rd right
74 ≤ LI < 80	4th right
80 ≤ LI < 84	5th right
84 ≤ LI < 88	6th right
88 ≤ LI < 92	7th right
92 ≤ LI < 95	8th right
95 ≤ LI < 100	9th right
LI = 100	10th right

Appendix B. Grasp names, pictures and categorization

No.	Name	Picture	Grasp type	Thumb position	Object's shape
1	Large diameter	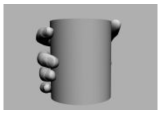	Power	Abducted	Bar large
2	Small diameter	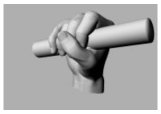	Power	Abducted	Bar small
3	Medium wrap	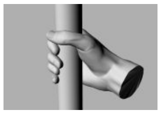	Power	Abducted	Bar large
4	Adducted thumb	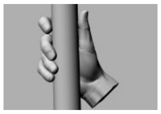	Power	Adducted	Bar large
5	Light tool	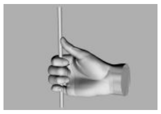	Power	Adducted	Bar small
6	Prismatic 4 finger	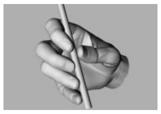	Precision	Abducted	Bar small
7	Prismatic 3 finger	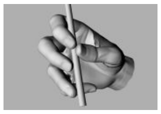	Precision	Abducted	Bar small
8	Prismatic 2 finger	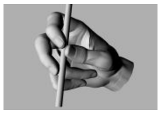	Precision	Abducted	Bar small
9	Palmar pinch	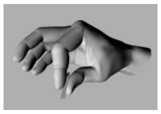	Precision	Abducted	Card
10	Power disk	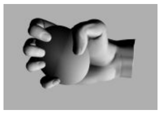	Power	Abducted	Disk
11	Power sphere	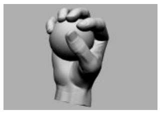	Power	Abducted	Sphere large
12	Precision disk	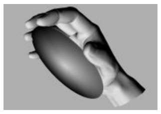	Precision	Abducted	Disk

13	Precision sphere	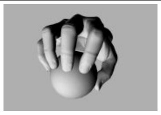	Precision	Abducted	Sphere large
14	Tripod	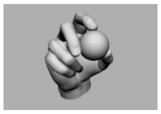	Precision	Abducted	Sphere small
15	Fixed hook	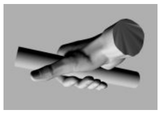	Power	Adducted	Bar large
16	Lateral	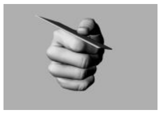	Intermediate	Adducted	Card
17	Index finger extension	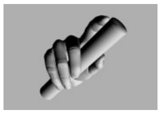	Power	Adducted	Bar large
18	Extension type	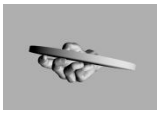	Power	Abducted	Disk
19	Distal type	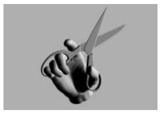	Power	Abducted	Scissors
20	Writing tripod	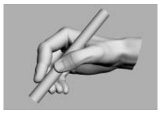	Precision	Abducted	Bar small
21	Tripod variation	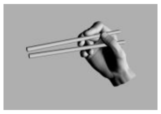	Intermediate	Abducted	Bar small
22	Parallel extension	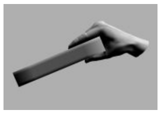	Precision	Adducted	Card
23	Adduction grip	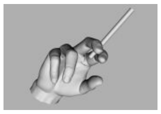	Intermediate	Abducted	Bar small
24	Tip pinch	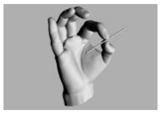	Precision	Abducted	Bar small
25	Lateral tripod	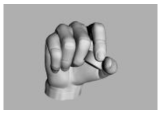	Intermediate	Adducted	Sphere small

26	Sphere<br>4 finger	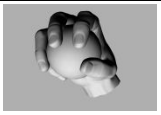	Power	Abducted	Sphere large
27	Quadpod	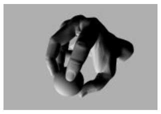	Precision	Abducted	Sphere small
28	Sphere<br>3 finger	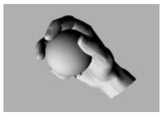	Power	Abducted	Sphere large
29	Stick	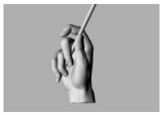	Intermediate	Adducted	Bar small
30	Palmar	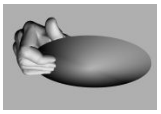	Power	Adducted	Disk
31	Ring	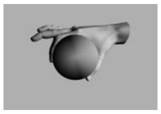	Power	Abducted	Sphere large
32	Ventral	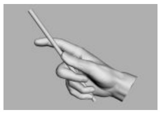	Intermediate	Adducted	Bar small
33	Inferior<br>pincer	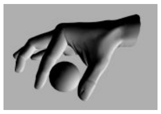	Precision	Abducted	Sphere small
